# Entropic measures of complexity in a new medical coding system

**DOI:** 10.1186/s12911-021-01485-y

**Published:** 2021-04-09

**Authors:** Jerome Niyirora

**Affiliations:** SUNY Polytechnic Institute, College of Health Sciences, Utica, NY USA

**Keywords:** Medical coding, Mapping, Complexity, Entropy

## Abstract

**Background:**

Transitioning from an old medical coding system to a new one can be challenging, especially when the two coding systems are significantly different. The US experienced such a transition in 2015.

**Objective:**

This research aims to introduce entropic measures to help users prepare for the migration to a new medical coding system by identifying and focusing preparation initiatives on clinical concepts with more likelihood of adoption challenges.

**Methods:**

Two entropic measures of coding complexity are introduced. The first measure is a function of the variation in the alphabets of new codes. The second measure is based on the possible number of valid representations of an old code.

**Results:**

A demonstration of how to implement the proposed techniques is carried out using the 2015 mappings between ICD-9-CM and ICD-10-CM/PCS. The significance of the resulting entropic measures is discussed in the context of clinical concepts that were likely to pose challenges regarding documentation, coding errors, and longitudinal data comparisons.

**Conclusion:**

The proposed entropic techniques are suitable to assess the complexity between any two medical coding systems where mappings or crosswalks exist. The more the entropy, the more likelihood of adoption challenges. Users can utilize the suggested techniques as a guide to prioritize training efforts to improve documentation and increase the chances of accurate coding, code validity, and longitudinal data comparisons.

## Introduction

Medical diagnoses and procedures are reported using standardized codes that are updated periodically to keep up with the latest clinical knowledge and practices. Transitioning from an old medical coding system to a new one can be challenging, especially when the two systems are significantly different. One such transition took place in the United States (US) in 2015 when the country switched from the 9th revision of the International Classification of Diseases (ICD) Clinical Modification (ICD-9-CM) to the 10th revision (ICD-10-CM). This newer revision was accompanied by a very different procedure coding system (PCS) (ICD-10-PCS), as compared to the ICD-9-CM procedure coding system (Volume 3, abbreviated here as Vol. 3). For example, each ICD-10-PCS procedure is made of 7 multi-axial characters where each axis encompasses up to 34 alphanumeric values [1]. This arrangement is a significant departure from the procedure code structure in ICD-9-CM Vol. 3, where all codes are numeric and can only be between 2 and 4 characters long. In 2015, ICD-10-PCS had about 72,000 procedure codes as compared to only about 4000 codes in ICD-9-CM Vol. 3. The diagnosis codes between these two revisions of ICD are also quite different. For example, all diagnosis codes in ICD-10-CM are alphanumeric and can be 3 to 7 characters long, whereas ICD-9-CM diagnosis codes are mostly numeric and can only be between 3 and 5 characters long. In 2015, there were about 14,500 diagnosis codes in ICD-9-CM as compared to about 69,800 codes in ICD-10-CM [2]. Given these differences, some analysts had predicted a costly and challenging transition from ICD-9-CM to ICD-10-CM/PCS [3]. Indeed, some of the feared problems did materialize after the changeover, such as the loss in productivity [4, 5], the lack of readiness of computer systems, the inability to find some ICD-9-CM concepts in the ICD-10-CM system, and difficulties mapping ICD-10-CM to other coding systems such as SNOMED-CT [6]. Some ICD-10-CM clinical classes were also found to have more coding deficiencies than others, such as the class of external causes of morbidity (V00-Y99) [7]. In one post-ICD-10 implementation audit, it was found that one of the most significant challenges for coders was selecting the correct character in the 3rd position (Root Operation), the 4th position (Body Part), and the 5th position (Approach) of an ICD-10-PCS code [8]. While little evidence exists to suggest that reimbursement was significantly impacted by the transition, in some practices, a statistical increase in the coding-related denials was noted [9]. A few of the post-transition qualitative studies concluded that training and education were critical in overcoming many of the previously anticipated challenges [6, 10]. Besides the US, other countries have also faced challenges while transitioning to new medical coding systems. The issues ranged from coding errors to discrepancy problems when the same condition was coded in both coding systems. For example, in one analysis [11], it was found that the Swiss transition from ICD-9 to ICD-10 resulted in the initial increase of the number of coding errors for co-morbidities, but, over time, the accuracy improved as the learning curve waned. In one Canadian study [12], the authors were interested in assessing the validity of ICD-10 codes after switching from ICD-9. While the authors did not find much difference in the validity of the codes from these two systems, the discrepancy was apparent for some conditions (e.g., HIV/AIDS, hypothyroidism, and dementia). The authors also observed that the quality of data had not yet improved in ICD-10 as originally expected.

Now that many countries are preparing to migrate from ICD-10 to ICD-11 [13], one can expect similar transition challenges to occur, as these two coding systems have different code structures [14], and the equivalence is at times lacking [15]. This research aims to introduce entropic measures to help users prepare for the migration to a new medical coding system by identifying and focusing preparation initiatives on clinical concepts with more likelihood of documentation deficiencies, coding errors, and longitudinal data comparison issues.

## Related work

Not many studies have considered how to quantify the complexity of codes between two medical coding systems. In some studies, the equivalence in the number and structure of the codes between two coding systems is considered, but without accompanying measures of the dissimilarity in the codes [15]. In a few studies, an attempt is made to address the complexity between two medical coding systems. For example, in Boyd et al. [16, 17], the authors proposed using the science of networks to evaluate the difficulties of transitioning from ICD-9-CM to ICD-10-CM in the US. The authors used general equivalence mappings (GEMs) to create graphs where diagnoses were nodes, and the relationships in the GEMs were edges. From their analysis, the authors derived directional motifs and identified convoluted mappings, where multiple medical codes from both coding systems shared complex, entangled, and non-reciprocal mappings. The authors concluded that clinical classes with convoluted mappings were more likely to be challenging to code and costly to implement after the changeover to the new medical coding system. Besides, these authors also anticipated that clinical classes with a high ratio of ICD-10-CM to ICD-9-CM codes were more likely to affect a smooth transition. Another study that considered the complexity of transitioning between two coding systems relates to the work of Chen et al. [18], where the authors leveraged Shannon’s entropy to develop a mapping framework between ICD-10 and ICD-11 coding systems. The authors proposed three entropy-based metrics of standardizing rate (SR), uncertainty rate (UR), and information gain (IG) to validate information changes between ICD-10 and ICD-11. The authors obtained the UR measure by $\sum _{i=1}^{M} p_i\log 1/p_i$, where *M* was the number of ICD-11 candidate codes for a single ICD-10 code, and $p_i$ was the probability of each ICD-11 code. In a special case of a uniform distribution, the authors suggested utilizing the average probability of 1/*M* to measure *UR*, which implied that $UR = \log M$. Among other conclusions, the authors recommended verifying ICD-10 codes with high UR measures as these codes were more likely to hinder a smooth transition to ICD-11.

## Contributions

This research complements previous studies highlighted in the Related Work section. For example, as in Chen et al. [18], this research proposes to apply Shannon’s entropy to study the complexity of the transition between two medical coding systems. Unlike in this previous study, the entropic measures in this research account for the variation in the alphabets of candidate codes. Besides, Shannon’s entropy is also used to create a measure of coding complexity that considers not only the number of candidate codes (as in the UR measure [18]) but also the number of combinations of these codes. As shown later, failure to account for the latter information may underestimate or overestimate the related coding complexity. It should also be mentioned that the proposed methods have an advantage over convoluted measures suggested in Boyd et al. [16, 17]. Unlike in the convoluted approach, where a code is classified as either being involved in a convoluted relationship or not, the proposed methods provide non-dichotomous complexity measures of each code.

## Materials and methods

### Methods

#### A motivating problem

It is imagined that a manager of a given medical care facility is preparing to transition from an old medical coding system *X* to a new medical coding system *Y*. The forward ($X\rightarrow Y$) and backward ($X\leftarrow Y$) mappings between *X* and *Y* are provided. The manager is unsure about employing these mappings to identify clinical concepts that are more likely to be challenging to translate into the new medical coding system. Some of the benefits of knowing this information include being able to formulate targeted training efforts for coding and clinical documentation to foster the validity of the data in the new coding system. Besides, understanding complex translations may help take the necessary steps to ensure longitudinal data comparisons. This research aims to suggest the techniques that the manager could use to solve this dilemma.

#### Model and assumptions

Given forward mappings ($X\rightarrow Y$), the old medical coding system *X* is termed the source system, while the new coding system *Y* is termed the target system. In the backward mappings ($X\leftarrow Y$), the *source* and *target* terminologies are reversed. For model development, only forward mappings are considered here since the backward mappings would obey the same logic. From the prescribed forward mappings ($X\rightarrow Y$), it is assumed that code $x\in X$ corresponds to *m* number of candidate codes $y \in Y$. This relationship, referred to here as a *map*, is symbolized as $x\rightarrow \left\{ y_1, y_2,\dots ,y_m\right\}$ or as in the following matrix form:

1 $$\begin{aligned} x\rightarrow \begin{bmatrix} a_{11} & a_{12} & \dots & a_{1n} \\ a_{21} & a_{22} & \dots & a_{2n} \\ \dots &\dots & \dots & \dots \\ a_{m1} & a_{m2} & \dots & a_{mn} \end{bmatrix} = \begin{bmatrix} y_{1} \\ y_{2} \\ \dots \\ y_{m} \end{bmatrix} \end{aligned}$$

where each code in the map $y_i$, for $i:1,\dots ,m$, has *n* fixed number of characters (also called alphabets) $a_{ij}$, for $j:1,\dots ,n$. If necessary, padding may be added to a particular code to ensure a constant length of *n* as this approach may simplify calculations. Each column represents an axis or simply a position of an alphabet in a code. The columns of a map are assumed to be independent. Each row of a map represents a valid code $y\in Y$. A set of more than one code in a map may be necessary to represent code $x \in X$. If $m = 0$, code *x* has no match in *Y*, which implies data loss in the new coding system. If $m = 1$, code $x\in X$ has a one-to-one relationship with code $y \in Y$. In this case, the coding complexity is expected to be zero since little surprise exists about what the new code should be. If $m > 1$, the coding complexity will be greater than zero as there is more than one candidate code in *Y*, thus more complexity and chances of coding or translation errors. In this research, a coding error is defined as the selection of a code where at least one alphabet is wrong or the selection of a set of codes where at least one of the codes is incorrect or missing. The expected coding complexity of a given clinical concept in *X* is characterized in terms of the uncertainty in the rows and columns of a map, which is measured here in bits units of Shannon’s entropy [19].

Two major sources of coding complexity are assumed here, namely source *A*, which captures the variation in the alphabets of a map, and source *B*, which relates to the combinations of the rows of a map. The entropy for source *A*, or *H*(*A*), is calculated as:

2 $$\begin{aligned} H(A) = -\sum _{j = 1}^{n}\sum _{i = 1}^{k_j} p_{ij}\log _2 p_{ij} \equiv \sum _{j=1}^{n} H(\varvec{a_j}) \end{aligned}$$

where $k_j\le m$ is the number of unique alphabets in column $\varvec{a_{j}}$ of matrix (1) and $p_{ij}$ is the probability of alphabet *i* in position *j*. The more the *H*(*A*) measure, the more requisite detailed documentation to express all the alphabets of a map. Likewise, the more the number of code alphabets that must be chosen separately, the more complex and time-consuming the coding.

Regarding source *B*, the corresponding entropy *H*(*B*) is obtained by:

3 $$\begin{aligned} H(B) =\log _2 (v) \end{aligned}$$

where $v = m_0 + \sum _{i=1}^{s}\prod _{j = 1}^{m - m_0}m_{ij}$. Here, *s* is the total number of possible scenarios and $m_0$ represents the number of stand-alone codes and, for a given scenario *i*, $m_{i1},\dots , m_{i(m-m_0)}$ denote the number of candidate codes in Y that must be combined to represent code $x\in X$. As before, *m* is the total number of candidate codes in a map. If a map only includes stand-alone codes, where no combinations of codes are required, Eq. 3 becomes *H*(*m*), which is comparable to the UR measure introduced in Chen et al. [18]. The more the *H*(*B*) measure, the more complex the coding due to the need for more coding memory and time, since more than one candidate code in the target system is going to be required to represent a single code from the source system. See Appendix A for more details on the derivation of Eqs. 2 and 3.

#### Implementation

It is recommended that both *H*(*A*) and *H*(*B*) entropic measures be normalized into $Z(\alpha )$ and $Z(\beta )$, as exemplified in Appendix A, to allow for the comparison and ranking of complexity from different sources. If *H*(*A*) and *H*(*B*) measures (or their normalized counterparts) are to be utilized to prepare for the transition (e.g., documentation improvement), they should be weighed using relevant empirical distribution (e.g., historical frequencies of codes in a given medical facility or general practice area). Accordingly, if, say, a particular facility never performs heart transplants, it shouldn’t have to spend too much training efforts on the documentation of this clinical concept. Algorithm 4.1 shows the steps that one can take to implement the suggested entropic methods.

##### Algorithm 4.1

Computing entropic measures

**Step 1:** Calculate *H*(*A*), the entropy of the columns of a map, to estimate the coding complexity due to the variation in the alphabets of the columns of a map.
**Step 2:** Calculate, *H*(*B*), the entropy of the rows of a map to estimate the coding complexity due to the uncertainty in the number of valid code representations in the map.
**Step 3:** Normalize *H*(*A*) and *H*(*B*) by centering these measures and then dividing them by their standard deviations. The normalized measures are symbolized here as $Z(\alpha )$ for *H*(*A*) and $Z(\beta )$ for *H*(*B*).
**Step 4:** If empirical data, based on historical visits or future forecasts, were available, one would adjust $Z(\alpha )$ and $Z(\beta )$ measures by multiplying them with the probability of a corresponding clinical concept.
**Step 5:** Use the adjusted or unadjusted entropic measures to prioritize transition initiatives between two medical coding systems.

### Materials

Algorithm 4.1 can be applied to evaluate entropic measures between any two medical coding systems, provided mappings or crosswalks exist. For demonstration, the 2015 US transition from ICD-9-CM to ICD-10-CM/PCS medical coding systems is considered. For a brief background, when the US was preparing to migrate from ICD-9-CM to ICD-10-CM/PCS, forward and backward general equivalence mappings (GEMs) were made available to users [2, 20]. A user could determine the number of candidate codes in the target system from these mappings, given a code in the source system. These files also allowed users to apply the given supplemental five digits codes (referred to as *flags*) to determine valid combinations of candidate codes in a map. For example, a flag code of 00000 or 10000 was used to represent a one-to-one relationship. The flag code of 00000 signified the exact equivalence, whereas a flag code of 10000 represented the approximate equivalence. If the relationship were one-to-many, the third character in the flag code would be 1 (instead of 0), and the fourth and fifth characters would specify combinations of candidate codes. The fourth character enumerated the number of scenarios, while the fifth character established the order that combinations were carried out in each scenario. The data used in this paper can be obtained directly from the CMS website at https://www.cms.gov/Medicare/Coding/ICD10/Archive-ICD-10-CM-ICD-10-PCS-GEMs. The 2015 GEMs, instead of the newer GEMs, are utilized here since they were the most updated mappings available to users to prepare for the transition from ICD-9-CM to ICD-10-CM/PCS in 2015.

### Demonstration

**Fig. 1:**
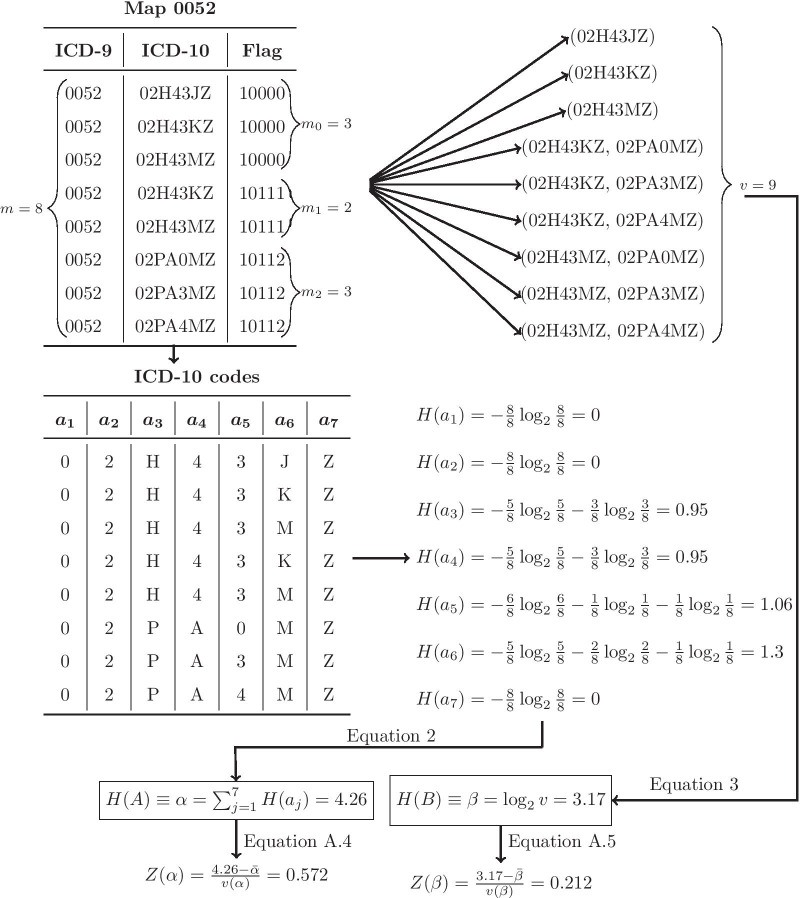
This figure depicts how to apply *Steps 1–3* of Algorithm 4.1 on map 0052. No empirical data were available to implement *Step 4* of this algorithm. *H*(*A*) is computed per Eq. 2. Equation A.3 is used to determine the number of valid representations *v* and *H*(*B*) is calculated per Eq. 3. The normalization of *H*(*A*) and *H*(*B*) follow Eqs. A.4 and A.5, respectively. The *UR* measure (proposed in Chen et al. [18]) of this map is obtained by $\log _2 (m)=\log _2 (8) = 3$. As compared to *H*(*B*), the UR measure slightly underestimates the complexity of map 0052. While no actual probabilities were available for *Step 4*, it still can be speculated that if the probability of implanting or replacing any electrodes in the ventricular coronary venous system were zero for a given medical facility, both the $Z(\alpha )$ and $Z(\beta )$ measures would be 0.572*0 = 0.212*0 = 0. The implementation of *Step 5* of this algorithm is discussed in “Results” section

Appendix E demonstrates a Python code to implement Algorithm 4.1. Figure 1 exhibits the application of this algorithm on map 0052. This map relates to an ICD-9-CM Vol.3 code of 00.52 for the *implantation or replacement of transvenous electrode into left ventricular coronary venous system*.

## Results

**Fig. 2:**
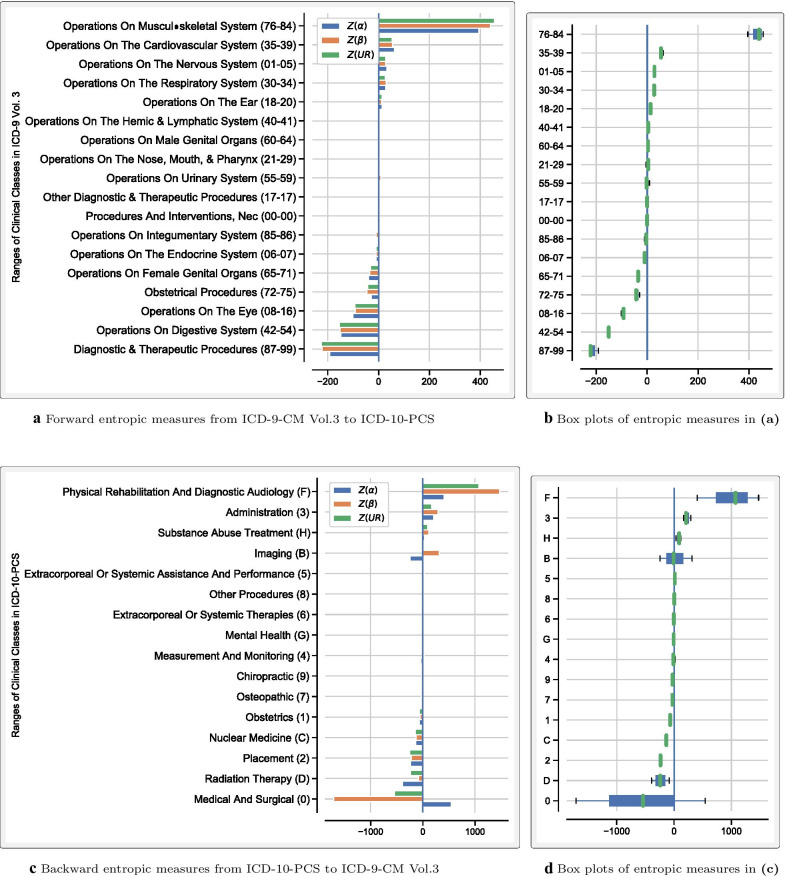
Forward and backward entropic measures between the procedure codes of ICD-9-CM Vol.3 and ICD-10-PCS. The x-axes represent the sum of $Z(\alpha )$, $Z(\beta )$, and *Z*(*UR*) entropic measures. **a**, **c** show clustered bar plots of the indicated clinical classes arranged from the least to the most sum of entropic measures. Negative values signify no information gained or lost information (on average) from the source system to the target system. Positive values suggest gained information. **b**, **d** display related box plots that may help visually assess the variation in the entropic measures in each clinical class. The wider the box, the more the interquartile range, thus the more variability in the measures. The tighter the box and whiskers, the more the measures agree

**Fig. 3:**
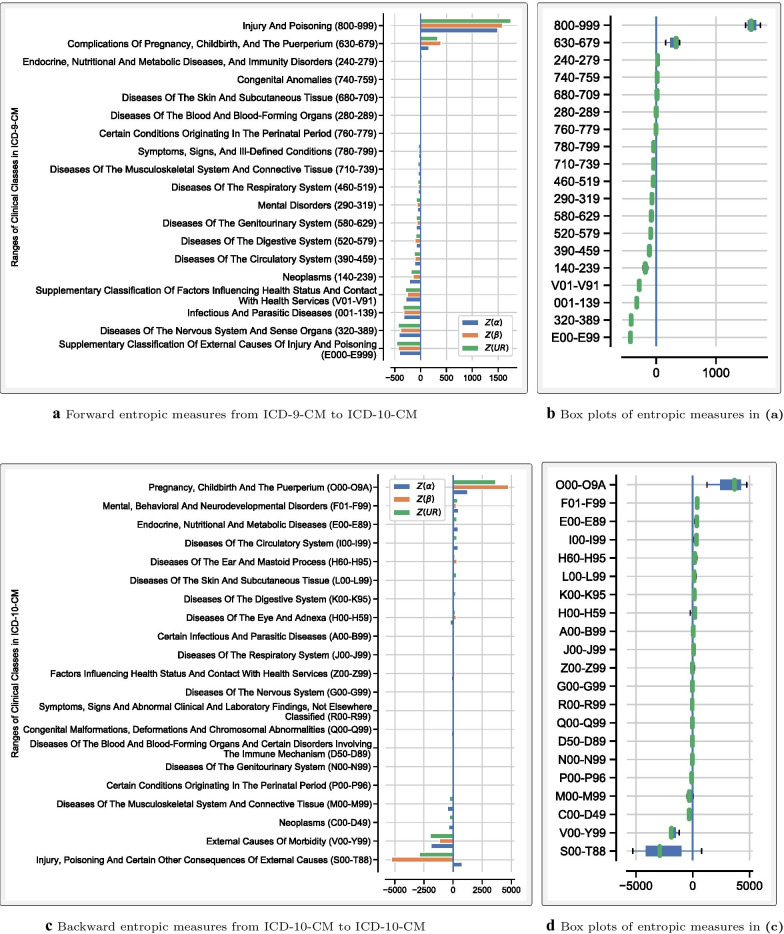
Forward and backward entropic measures between the diagnosis codes of ICD-9-CM and ICD-10-CM. The x-axes represent the sum of $Z(\alpha )$, $Z(\beta )$, and *Z*(*UR*) entropic measures. **a**, **c** show clustered bar plots of the indicated clinical classes arranged from the least to the most sum of entropic measures. Negative values signify no information gained or lost information (on average) from the source system to the target system. Positive values suggest gained information. **b**, **d** display related box plots that may help visually assess the variation in the entropic measures in each clinical class. The wider the box, the more the interquartile range, thus the more variability in the measures. The tighter the box and whiskers, the more the measures agree

**Fig. 4:**
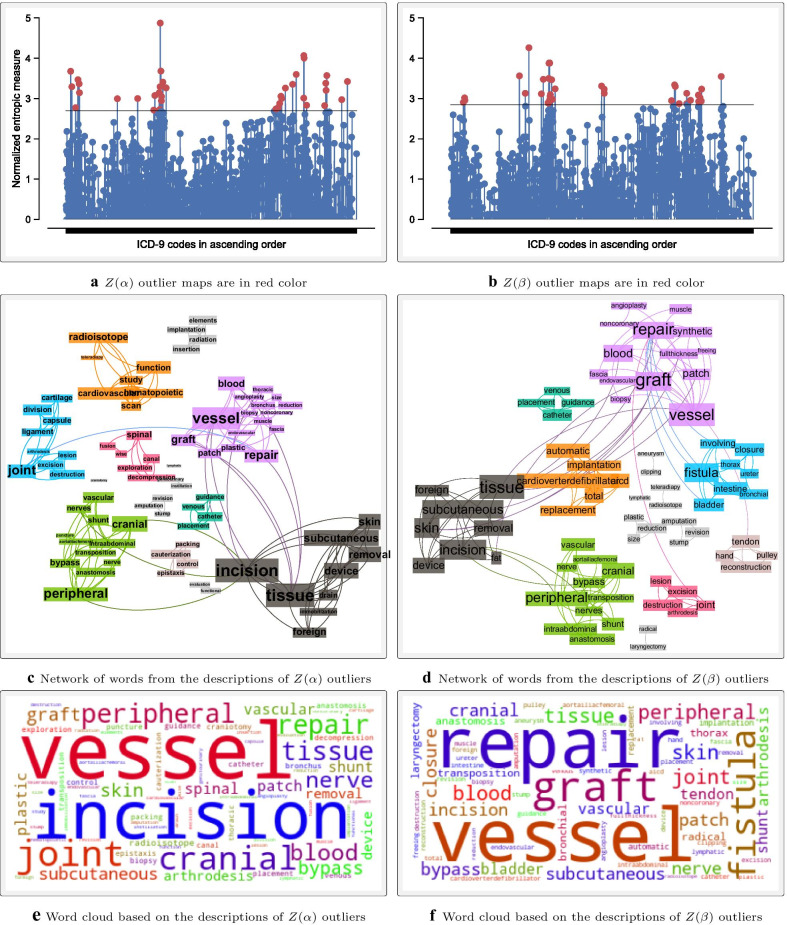
An example of outlier and pattern analysis based on forward mapping from ICD-9-CM Vol.3 and ICD-10-PCS. Figure **a**, **b** show red dots for outlier maps where $Z(\alpha )$ and $Z(\beta )$ scores are greater than the chosen threshold. For illustration purposes, the threshold were determined (2.7 for $Z(\alpha )$ and 2.85 for $Z(\beta )$, so that only the top 1% of the cases are isolated. The maps that were isolated are shown in Appendices C and D. **c** illustrates a network of words in the descriptions of $Z(\alpha )$ outlier maps while **d** portrays a similar network for $Z(\beta )$ outlier maps. The corresponding word clouds are respectively shown in **e**, **f**

Algorithm 4.1 was applied to both forward and backward GEMs between ICD-9-CM and ICD-10-CM/PCS. Tables 1 and 2 display the corresponding descriptive statistics for *H*(*A*), *H*(*B*), and *UR* entropic measures. Codes without match in the target system were excluded from these statistics. For comparison purposes, the normalization of the *UR* measure [18] is symbolized as *Z*(*UR*). To implement **Step 5** of Algorithm 4.1, clinical concepts were ranked by their entropic measures. Figures 2 and 3 show ranked clinical classes from the least to the most sum of $Z(\alpha )$, $Z(\beta )$, and *Z*(*UR*) measures. The classes in these figures were also ranked separately using each entropic measure. As expected, the resulting rankings based on $Z(\alpha )$, $Z(\beta )$, and *Z*(*UR*) measures were not always consistent. To assess how much the rankings of these entropic measures agreed, the Kendall tau correlation coefficients were assessed, and the results are presented in Table 3. The closer to 1 the Kendall tau value (the greener the color), the more the given entropic measures agreed. An alternative approach to implementing **Step 5** of Algorithm 4.1 is performing outlier and pattern analysis and then segregate concepts that should receive more attention during the transition. An example of how such an analysis may be conducted is shown in Fig. 4. To extract thematic descriptions of the outlier maps, network analysis techniques suggested in Niyirora and Aragones [21] were applied after removing stopwords [22] and residual words (e.g., other, unspecified, etc.). Communities of words in Fig. 4c, d (distinguished by different colors) were isolated using the modularity algorithm in Gephi [23]. To gauge the frequency (or significance) of words in the outlier maps, a word cloud analysis was undertaken, where the bigger the word meant, the more significant the word (see Fig. 4e, f).

**Table 1:** Descriptive statistics of the *H*(*A*), *H*(*B*), and *UR* entropic measures between ICD-9-CM Vol.3 and ICD-10-PCS

	Forward mapping			Backward mapping		
	From ICD-9-CM Vol.3 to ICD-10-PCS			From ICD-10-PCS to ICD-9-CM Vol.3		
	H(A)	H(B)	UR	H(A)	H(B)	UR
Count	3672	3672	3672	71924	71924	71924
Mean	2.76	3.03	2.74	0.09	0.25	0.13
Std	1.92	2.16	1.85	0.32	0.84	0.40
Min	0.00	0.00	0.00	0.00	0.00	0.00
25%	1.00	1.58	1.44	0.00	0.00	0.00
50%	2.58	2.58	2.58	0.00	0.00	0.00
75%	4.00	4.39	3.91	0.00	0.00	0.00
Max	10.95	13.53	10.22	3.46	7.50	3.46

**Table 2:** Descriptive statistics of the *H*(*A*), *H*(*B*), and *UR* entropic measures between ICD-9-CM and ICD-10-CM

	Forward mapping			Backward mapping		
	From ICD-9-CM to ICD-10-CM			From ICD-10-CM to ICD-9-CM		
	*H*(*A*)	*H*(*B*)	*UR*	*H*(*A*)	*H*(*B*)	*UR*
Count	14567	14567	14567	69823	69823	69823
Mean	0.52	0.30	0.33	0.30	0.07	0.12
Std	1.26	0.69	0.72	1.01	0.28	0.36
Min	0.00	0.00	0.00	0.00	0.00	0.00
25%	0.00	0.00	0.00	0.00	0.00	0.00
50%	0.00	0.00	0.00	0.00	0.00	0.00
75%	0.00	0.00	0.00	0.00	0.00	0.00
Max	13.10	9.06	9.06	7.31	3.58	3.58

**Table 3:** Kendall tau correlation among the rankings of clinical classes using the normalized entropic measures ($Z(\alpha )$, $Z(\beta )$, and *Z*(*UR*))

				Forward mapping			Backward mapping		
				$Z(\alpha )$	$Z(\beta )$	*Z*(*UR*)	$Z(\alpha )$	$Z(\beta )$	*Z*(*UR*)
ICD-9-CM Vol 3.	$<>$	ICD-10-PCS	$Z(\alpha )$		0.99	0.99		0.97	0.97
			$Z(\beta )$	0.99		1.00	0.97		1.00
			*Z*(*UR*)	0.99	1.00		0.97	1.00	
ICD-9-CM	$<>$	ICD-10-CM	$Z(\alpha )$		0.85	0.87		0.57	0.88
			$Z(\beta )$	0.85		0.98	0.57		0.66
			*Z*(*UR*)	0.87	0.98		0.88	0.66	

The symbol $<>$ is used to signify mapping between the indicated medical coding systems

## Discussion

In 2015, ICD-10-PCS had a significantly greater number of procedure codes (n = 71,924), as compared to ICD-9-CM Vol.3 (n = 3,672) (see Table 1). Equally, Table 2 shows more diagnosis codes for ICD-10-CM vis-à -vis ICD-9-CM. This fact alone implies that more specific information was likely to be gained by migrating from ICD-9-CM to ICD-10-CM/PCS, assuming complete clinical documentation and accurate coding. The mean statistics in these tables reveal that all the entropic measures are higher in the forward mappings than the backward mappings. This revelation further certifies that, on average, more information was gained in ICD-10-CM/PCS as compared to ICD-10-CM. The quartile statistics with a value of zero suggest the minimum percentage of one-to-one mapping from the source system (e.g., a 75% quartile of zero indicates that at least 75% of codes in the source system has a one-to-one relationship with the target system). A one-to-one relationship implies that no information is gained since $log (1) = 0$. In other words, one-to-one codes may structurally look different, but if they represent the same clinical concept, then no information is gained. To a computer, a one-to-one mapping is a simple translation, but, of course, to a human coder, more complicated code structures may be more challenging to extract and translate.

The scale of the information gained (or lost) between ICD-9-CM and ICD-10-CM/PCS can be appreciated by clinical classes depicted in Figs. 2 and 3. For example, Fig. 2a indicates that in the procedural forward mappings, the most information was gained in the class of the *Operations on Musculoskeletal System (76–84)*. The related box plot in Fig. 2b suggests that all three entropic measures relatively agreed on the characterization of class 76–84, given the small interquartile range. For diagnoses, Fig. 3a suggests that the class of the *Injury and Poisoning (800–999)* carried more forward information in ICD-10-CM followed by the class of *Pregnancy and Childbirth (630–679)*. Remarkably, Fig. 3c implies that an ICD-10-CM class related to Pregnancy and Childbirth (O00-O9A) also resulted in backward information gain in ICD-9-CM. These conflicting results are due to the convoluted nature of the mappings between these two medical coding systems [16].

From Figs. 2 and 3, one notices that some clinical classes have negative entropic measures. This observation implies that little, or no information, would be gained in the target system. For example, Fig. 2a indicates that for the procedure class of the *Diagnostic & Therapeutic Procedures (87–99)*, not much forward information was gained in ICD-10-PCS. Likewise, little, or no backward information was gained in ICD-9-CM Vol. 3 about the procedure class of *Medical and Surgical (0)* (see Fig. 2c). However, the entropic measures somewhat disagree on the latter suggestion, given a large inter-quartile range of class (0) in Fig. 2d. Regarding diagnosis codes, Fig. 3a suggests that little, or no information, was gained in ICD-10-CM about the ICD-9-CM class of *Supplementary Classification Of External Causes Of Injury And Poisoning (E000-E999)*. In an apparent contraction, Fig. 3c points to the lack of information gained in the backward mapping about ICD-10-CM classes of *Injury, poisoning and certain other consequences of external causes (S00-T88)* and *External causes of morbidity (V00-Y99)*. Again, this ambiguity results from the complex relationship between ICD-9-CM and ICD-10-CM/PCS coding systems [16].

It is noteworthy that, despite a greater number of codes in ICD-9-CM/PCS, the backward max statistics in both Tables 1 and 2 are not zero. This finding implies that, for some clinical concepts, ICD-9-CM captured more information vis-à-vis ICD-10-CM/PCS (e.g., class (F) in Fig. 2c and class (O00-O9A) in Fig. 3c). The implication is that some ICD-9-CM information was lost in ICD-10-CM/PCS, which created issues with longitudinal data comparisons. This dilemma also likely produced problems with verifying ICD-10-CM/PCS codes’ validity, especially for classes where the information was gained in both forward and backward mappings (bidirectional) (e.g. in the pregnancy and childbirth clinical class). Additional challenges resulting from the bidirectional information gain include conflicting documentation requirements, primarily if the new coding system collects different clinical information than what is commonly documented. Naturally, coding errors are likely to ensue if clinical documentation is lacking or inconsistent.

To prepare for the transition to a new medical coding system, the user can utilize the proposed entropic measures as a guide to orient training efforts. To this end, clinical classes can be ranked to gauge where most information is likely to be gained or lost. Of course, the user would have more confidence if the rankings of these entropic measures agreed. Regarding the transition from ICD-9-CM to ICD-10-CM/PCS, the proposed methods tend to provide similar rankings. This fact is particularly true in the forward mappings from ICD-9-CM Vol.3 to ICD-10-PCS, where, as highlighted in Table 3, Kendall tau correlation coefficients between the methods are either 1 or very close to 1. However, in some instances, such as in the backward mappings from ICD-10-CM to ICD-9-CM (see Table 3), the methods may disagree. Significant differences between *H*(*A*) (the entropy of the alphabets or columns of a map) and *H*(*m*) (the entropy of the rows of a map) typically cause this disagreement. The entropic measures will always agree in cases of a single candidate code in the map ($m = 1$) since the entropy is zero for all measures. As the number of candidate codes *m* increases, *H*(*m*) increases as expected, which should also increase *H*(*A*). While such a mutual increase in both *H*(*A*) and *H*(*m*) occurs in most maps, a few maps exhibit more variation in the codes’ alphabets relative to the corresponding number of candidate codes or vice versa. An example here is map 721 (Low forceps operation with episiotomy) where *H*(*A*) = 6, but *H*(*m*) = 0.5 since there are only two candidate codes. In this map, *H*(*B*)–the entropy of the valid combinations (*v*) of *m* candidate codes–is zero since $v = 1$. In map 7392 (Replacement of prolapsed umbilical cord), the opposite divergence exists. There are more codes ($m = 3$) relative to the corresponding variation in the alphabets. As a result, *H*(*m*) = 1.59 whereas *H*(*A*) = 0.92. Besides the disparity between *H*(*m*) and *H*(*A*), *H*(*B*) and *H*(*m*)–two entropic methods that mostly agree–may also significantly diverge when there is a significant difference in the number of candidate codes *m* and the number of valid combinations *v*. Examples include map 0050 (Implantation of cardiac resynchronization pacemaker without mention of defibrillation, total system [CRT-P]) where $v = 216$ but $m = 16$ and map 688 (Pelvic evisceration) where $v = 2$ but $m = 16$. Regardless of the source, a divergence in the entropic measures’ rankings complicates implementing the proposed methods in actual settings. Unless one method proved superior to others, clinical concepts or classes where the rankings of entropic measures significantly disagree should be audited by medical providers and coding professionals. Subsequently, training efforts for clinical documentation and medical coding should be adjusted as appropriate. Given this recommendation, during the transition from ICD-9-CM to ICD-10-CM/PCS, audits of clinical classes 0, B, and F (in Fig. 2b) and O00–O9A and ST00–T88 (in Fig. 2d) would have been necessary to ascertain any transition challenges and training needs.

Besides ranking maps or clinical classes by their entropic measures, the user may also prioritize transition efforts from outlier and pattern analysis. That is, instead of working with predefined clinical classes, the user would try to assess the impact of the transition using major themes or ontological groups from the descriptions of outlier maps. Many thematic analysis [24] and ontological learning methods [25, 26] are applicable here. For demonstration purposes, a simple graph was constructed and patterns were examined using network algorithms [21, 27] (see Fig. 4). For example, a close examination of Fig. 4c, d reveal a collection of terms that relate to the vascular, skeletal, integumentary, and cardiac body systems. In terms of the eigenvector centrality, the most central words were tissue, graft, subcutaneous, skin, repair, and incision. Combining these keywords, one may conclude that the procedures for the *musculoskeletal, integumentary, and cardio-vascular systems* likely involved significant complex coding in ICD-10-PCS, a deduction that is consistent with the results in Fig. 2a.

## Conclusion

Transitioning from an old medical coding system to a new one can be challenging, especially when the two coding systems are significantly different. This research aimed to propose methods that could help users prepare for the transition by identifying and focusing preparation initiatives on clinical concepts with more likelihood of transition challenges. To this end, two entropic measures of coding complexity were introduced. The first measure was a function of the variation in the map’s alphabets, and the second measure was based on the possible number of valid combinations of candidate codes in a map. The primary assumption was that the more entropy, the more likelihood of coding errors. So, more prudent documentation was recommended for clinical concepts with high rankings of entropic measures, not only to increase the chances of accurate coding but also code validity and longitudinal data comparisons. It was also recommended that the resulting entropic measures be normalized and adjusted by the probability of a given code before isolating clinical concepts of interest. Medical professionals should conduct audits to ascertain transition challenges and training needs, particularly in the instances of diverging entropic measures. The proposed techniques are suitable for establishing coding complexity between any two medical coding systems, provided mappings or crosswalks exist. A demonstration of how to implement the proposed entropic measures was carried out using the 2015 forward and backward mappings between ICD-9-CM and ICD-10-CM/PCS.

## Limitations and future research

A central conjecture of this research was that clinical concepts with more entropic measures were more likely to result in a more challenging transition. The justification of this assumption emanated from the fact that more entropic measures meant more variation in the codes, thus necessitating more prudent documentation and coding. This assumption may be violated if documentation is already complete and an experienced coder knows shortcuts to circumvent the new coding complexity. Accordingly, a medical record review may be necessary to ensure that the apparent complexity from the entropic measures actually exists. Besides, the topic of correlation between code validity and related entropic measures was not explored in this research. A medical record review may also be necessary to see if any lack of validity in the codes is explained by reasons other than the entropic measures. Other relevant topics could not be considered in this research without additional data. For example, the question of how a new medical coding system could affect reimbursement was not entrained. Also, the topic of how coding guidelines and conventions may contribute to coding errors in the new system was not discussed. Future research goals include considering these other topics, especially as they relate to the upcoming (or ongoing for some countries) transition from ICD-10 to ICD-11. Future research efforts will also include applying the proposed methods to the mapping between ICD-10 (or ICD-11) and SNOMED CT.

## Data Availability

The data used in this paper can be obtained directly from the CMS website at https://www.cms.gov/Medicare/Coding/ICD10/Archive-ICD-10-CM-ICD-10-PCS-GEMs. These data are also included in the supplemental materials. The Python code used here is also included in E.

## Appendix A

### Calculating entropic measures of a map

#### Calculating Shannon’s entropy

Given a source S with *m* events and probabilities $p_1, p_2,\dots , p_m$ such that $\sum _{i= 1}^{m} p_i = 1$, the entropy *H* of the source *S* is computed as $H(S) = -\sum _{i = 1}^{m} p_i\log _2 p_i$, where $0\log 0 = 0$. The subscript of 2 under the $\log$ symbol signifies bits units. The entropy of two independent sources *S* and *T* is given by $H(S, T) = H(S) + H(T)$. If the sources are dependent, the conditional entropy is used to obtain *H*(*S*, *T*), as follows $H(S, T) = H(S) + H(T|S) = H(T) + H(S|T)$ [19].

#### Using Shannon’s entropy to estimate coding complexity

As noted in the main text, two major sources of coding complexity considered here are source *A*, which relates to the events of the alphabets in each column $\varvec{a_{j}}$ of the map, for $j:1,\dots ,n$, and source *B*, which relates to the events of the combinations of the rows (or codes) of the same map. Shannon’s entropy of column $\varvec{a_{j}}$, $H(\varvec{a_{j}})$, is given by $H(\varvec{a_{j}}) = -\sum _{i = 1}^{k_j} p_{ij}\log _2 p_{ij}$ where $k_j\le m$ is the number of unique alphabets in column $\varvec{a_{j}}$ and $p_{ij}$ is the probability of alphabet *i* in position *j*. From Eq. 2, it was indicated that $H(A) = \sum _{j=1}^{n} H(\varvec{a_j}) = -\sum _{j = 1}^{n}\sum _{i = 1}^{k_j} p_{ij}\log _2 p_{ij}$. A more refined measure of *H*(*A*) is possible to reflect the fact that the position of an alphabet in a code may carry a different weight of a coding error. For example, in ICD-10, a coding error where the first character is incorrect is typically much worse than a coding error where the last character is wrong since the first alphabet tends to serve as a root node for the classification of clinical concepts. Accordingly, a weighing scheme can be devised to account for the relative influence of the position of an alphabet in a code. If $w_j$ were the weight of position *j* in the code, the resulting weighted average entropy of source A, $H(\bar{A})$, may look like this:

A.1 $$\begin{aligned} H(\bar{A})= \frac{\sum _{j = 1}^{n} \log _2\left( \prod _{i=1}^{k_j} p_{ij}^{- p_{ij}}\right) ^{w_j}}{ \sum _{j = 1}^{n} w_j} \end{aligned}$$

where, as before, $k_j\le m$ is the number of unique alphabets in column $\varvec{a_{j}}$ and $p_{ij}$ is the probability of alphabet *i* in position *j*, for $j:1,\dots ,n$. The proof of Eq. A.1 follows from a logarithmic rules of $\log (x.y) = \log (x) + \log (y)$ and $a.\log (x) = log(x)^a$. Hence, it follows that $-\sum _{j = 1}^{n}\sum _{i = 1}^{k_j} p_{ij}\log p_{ij} = -\sum _{j = 1}^{n}\sum _{i = 1}^{k_j} \log p_{ij}^{- p_{ij}} = \sum _{j = 1}^{n} \log \left( \prod _{i=i}^{k_j} p_{ij}^{- p_{ij}}\right)$. After weighing each column, it is evident that $\sum _{j = 1}^{n} w_j \cdot \log \left( \prod _{i=i}^{k_j} p_{ij}^{- p_{ij}}\right)$ is equivalent to $\sum _{j = 1}^{n} \log \left( \prod _{i=1}^{k_j} p_{ij}^{- p_{ij}}\right) ^{w_j}$. The denominator in Eq. A.1 allows for the calculation of the average of the weighted entropy.

As for source B, it was indicated in Eq. 3 that $H(B) =\log (v)$. The proof of this equation follows from the fact that, under the assumption of a uniform distribution, each valid representation is equally likely, with probability 1/*v*. Then by Shannon’s entropy, $H(B) = -\sum _{j= 1}^{v} \frac{1}{v} \log \left( \frac{1}{v}\right) = -\frac{v}{v} (\log 1 -\log (v)) = log(v)$. If all *m* number of candidate codes in a map have a one-to-one relationship with code *x*, then all these candidate codes are considered stand-alone and don’t have to be combined to form a valid representation of code *x*. In such case, the number of valid representations *v* equals the number of stand-alone $m_0$, which also equals the number of candidate codes *m*. This means that:

A.2 $$\begin{aligned} H(B) = \log (v) \equiv \log (m) \equiv \log (m_0) \end{aligned}$$

Equation A.2 is comparable to a case of the UR measure in Chen et al. [18]. If a map includes some stand-alone codes and other codes that need to be combined under different scenarios to make valid representations of code *x*, then *v* is obtained by:

A.3 $$\begin{aligned} v = m_0 + \sum _{i=1}^{s}[(m_1)(m_2)\dots (m_{m - m_0})]_i \equiv m_0 + \sum _{i=1}^{s}\prod _{j = 1}^{m - m_0}m_{ij} \end{aligned}$$

where *i* represents scenario *i* out of *s* total number of scenarios. Here, $m_0$ is again the number of stand-alone codes and ${m - m_0}$ is the number of codes that must be combined in sequential order of their index, as a set, to represent the old code *x*. That is, for a given scenario *i*, $m_{i1}$ is the number of codes that must be sequenced first, followed by $m_{i2}$, the total number of codes that must be sequenced second followed by $m_{i3}$, the total number of codes that must be sequenced third, and so on until $m_{i(m-m_0)}$. If $m = m_0$, then, as indicated in Eq. A.2, $v = m_0$. The justification of Eq. A.3 comes from the fact that if a map has some stand-alone codes, then there are ${m_0\atopwithdelims ()1} = \frac{m_0!}{1!(m_0-1)!} = m_0$ possibilities of choosing one stand-alone code at random. If a map has at least one scenario, then for each scenario *i*, there are ${m_{i1}\atopwithdelims ()1}.{m_{i2}\atopwithdelims ()1}\dots {m_{i(m - m_0)}\atopwithdelims ()1} = (m_{i1})(m_{i2})\dots (m_{i(m - m_0)})$ possibilities of choosing one sequence of codes from $m_{i1}$ to $m_{i(m - m_0)}$. Since stand-alone codes don’t have to be combined with any other codes, then, for a map with a single scenario, the number of valid representations of code *x* is given by $v = m_0 + (m_1)(m_2)\dots (m_{m - m_0})$ (review the example in Fig. 1). If a map had more than one scenario, the total number of valid representations of code *x* would follow Eq. A.3.

#### Normalizing the entropic measures

Assuming that $H(A) \equiv \alpha$ and $H(B)\equiv \beta$, the normalized entropic scores can be obtained this way:

A.4 $$\begin{aligned} Z(\alpha )&= \frac{\alpha - \bar{\alpha }}{var(\alpha )} \end{aligned}$$

A.5 $$\begin{aligned} Z(\beta )&=  \frac{\beta - \bar{\beta }}{var(\beta )} \end{aligned}$$

where $\bar{\alpha }$ is the average of $\alpha$ measures from all maps and $\bar{\beta }$ is the average of $\beta$ measures from all maps. The symbol *var*() signifies the variance function.

## Appendix B

### Top 30 maps in the forward mapping from ICD-9-CM to ICD-10-CM

See Table 4.

**Table 4:** Top 30 maps in the forward mapping from ICD-9-CM to ICD-10-CM, ranked by their sum of $Z(\alpha )$, $Z(\beta )$, and *Z*(*UR*), from most to least total score

Map	Map description	$Z(\alpha )$	$Z(\beta )$	*Z*(*UR*)	Total score
V5412	Aftercare for healing traumatic fracture of lower arm	7.99	12.72	12.15	32.87
V5416	Aftercare for healing traumatic fracture of lower leg	7.95	12.47	11.90	32.31
V5411	Aftercare for healing traumatic fracture of upper arm	6.74	10.80	10.31	27.86
99529	Unspecified adverse effect of other drug, medicinal and biological substance	7.44	10.19	9.72	27.36
V5413	Aftercare for healing traumatic fracture of hip	7.14	10.19	9.72	27.05
V5417	Aftercare for healing traumatic fracture of vertebrae	6.78	10.00	9.54	26.32
V5415	Aftercare for healing traumatic fracture of upper leg	7.09	9.80	9.35	26.23
9895	Toxic effect of venom	6.12	10.10	9.63	25.85
99811	Hemorrhage complicating a procedure	10.01	8.08	7.70	25.78
99812	Hematoma complicating a procedure	10.01	8.08	7.70	25.78
V5419	Aftercare for healing traumatic fracture of other bone	7.73	9.11	8.69	25.53
29289	Other specified drug-induced mental disorders	6.43	9.34	8.91	24.68
9982	Accidental puncture or laceration during a procedure, not elsewhere classified	9.94	6.14	5.84	21.92
73382	Nonunion of fracture	6.45	6.90	6.56	19.91
9050	Late effect of fracture of skull and face bones	5.45	6.83	6.50	18.78
9947	Asphyxiation and strangulation	4.53	7.25	6.90	18.68
98989	Toxic effect of other substance, chiefly nonmedicinal as to source, not elsewhere classified	5.14	6.47	6.16	17.77
24980	Secondary diabetes mellitus with other specified manifestations, not stated as uncontrolled, or unspecified	4.93	6.47	6.16	17.57
9880	Toxic effect of fish and shellfish eaten as food	5.19	6.23	5.92	17.34
9823	Toxic effect of other chlorinated hydrocarbon solvents	4.36	6.55	6.23	17.14
9063	Late effect of contusion	6.13	5.63	5.35	17.10
8065	Open fracture of lumbar spine with spinal cord injury	5.13	5.63	5.92	16.68
9057	Late effect of sprain and strain without mention of tendon injury	6.16	5.38	5.11	16.64
E959	Late effects of self-inflicted injury	4.82	5.85	5.56	16.22
E9990	Late effect of injury due to war operations	4.81	5.85	5.56	16.21
64131	Antepartum hemorrhage associated with coagulation defects, delivered, with or without mention of antepartum condition	3.72	6.31	6.01	16.04
8064	Closed fracture of lumbar spine with spinal cord injury	4.49	5.63	5.92	16.03
9064	Late effect of crushing	5.50	5.38	5.11	15.98
986	Toxic effect of carbon monoxide	4.56	5.85	5.56	15.97
73395	Stress fracture of other bone	5.38	5.38	5.11	15.87

The map id and description correspond to ICD-9-CM code and description, respectively

## Appendix C

### Outliers maps of $Z(\alpha )$ where $Z(\alpha ) > 2.7$ (about top 1% of the maps)

See Table 5.

**Table 5:** Outlier maps arranged in the decreasing order of the $Z(\alpha )$ scores. As before, *m* is the number of candidate codes in a map whereas *v* is the number of valid representations in a map

Map	Map description	*m*	*v*	${Z(\alpha )}$
3929	Other (peripheral) vascular shunt or bypass	1191	1191	4.87
8605	Incision with removal of foreign body or device from skin and subcutaneous tissue	415	415	4.06
8609	Other incision of skin and subcutaneous tissue	335	335	4.00
3950	Angioplasty of other non-coronary vessel(s)	1196	1196	3.68
0109	Other cranial puncture	34	34	3.68
843	Revision of amputation stump	349	349	3.60
9301	Functional evaluation	148	148	3.57
0404	Other incision of cranial and peripheral nerves	327	327	3.47
9788	Removal of external immobilization device	98	98	3.42
3979	Other endovascular procedures on other vessels	689	689	3.41
9223	Radioisotopic teleradiotherapy	768	768	3.38
046	Transposition of cranial and peripheral nerves	378	378	3.37
8382	Graft of muscle or fascia	440	440	3.35
0124	Other craniotomy	111	111	3.29
3926	Other intra-abdominal vascular shunt or bypass	720	720	3.29
409	Other operations on lymphatic structures	510	510	3.27
8196	Other repair of joint	313	313	3.26
9227	Implantation or insertion of radioactive elements	268	268	3.21
3897	Central venous catheter placement with guidance	48	320	3.15
0474	Other anastomosis of cranial or peripheral nerve	350	350	3.15
3821	Biopsy of blood vessel	699	699	3.08
8120	Arthrodesis of unspecified joint	582	582	3.06
3958	Repair of blood vessel with unspecified type of patch graft	421	421	3.05
8604	Other incision with drainage of skin and subcutaneous tissue	281	281	3.01
3348	other repair and plastic operations on bronchus	288	288	3.00
8129	Arthrodesis of other specified joints	552	552	3.00
2103	Control of epistaxis by cauterization (and packing)	10	8	3.00
9649	Other genitourinary instillation	5	5	2.97
3956	Repair of blood vessel with tissue patch graft	402	402	2.95
8100	Spinal fusion, not otherwise specified	282	282	2.88
8683	Size reduction plastic operation	340	340	2.84
9205	Cardiovascular and hematopoietic scan and radioisotope function study	54	54	2.83
0309	Other exploration and decompression of spinal canal	70	70	2.78
8080	Other local excision or destruction of lesion of joint, unspecified site	345	345	2.77
8040	Division of joint capsule, ligament, or cartilage, unspecified site	210	210	2.74
3925	Aorta-iliac-femoral bypass	320	320	2.73
3805	Incision of vessel, other thoracic vessels	78	78	2.71

The map id and description correspond to ICD-9-CM Vol. 3 code and description, respectively

## Appendix D

### Outliers maps of $Z(\beta )$ where $Z(\beta ) > 2.85$ (about top 1% of the maps)

See Table 6.

**Table 6:** Outlier maps arranged in the decreasing order of the $Z(\beta )$ scores

Map	Map description	*H*(*B*)	*UR*	*m*	*v*	${Z(\beta )}$
3473	Closure of other fistula of thorax	10.95	7.92	243	1977	4.26
3950	Angioplasty of other non-coronary vessel(s)	10.22	10.22	1196	1196	3.88
3929	Other (peripheral) vascular shunt or bypass	10.22	10.22	1191	1191	3.88
304	Radical laryngectomy	9.61	5.81	56	784	3.57
9223	Radioisotopic teleradiotherapy	9.58	9.58	768	768	3.55
3926	Other intra-abdominal vascular shunt or bypass	9.49	9.49	720	720	3.50
3821	Biopsy of blood vessel	9.45	9.45	699	699	3.48
3979	Other endovascular procedures on other vessels	9.43	9.43	689	689	3.47
8120	Arthrodesis of unspecified joint	9.18	9.18	582	582	3.34
5684	Closure of other fistula of ureter	9.13	6.13	70	560	3.31
8129	Arthrodesis of other specified joints	9.11	9.11	552	552	3.30
409	Other operations on lymphatic structures	8.99	8.99	510	510	3.24
8687	Fat graft of skin and subcutaneous tissue	8.98	6.04	66	506	3.24
5783	Repair of fistula involving bladder and intestine	8.98	6.13	70	505	3.23
3342	Closure of bronchial fistula	8.78	7.03	131	440	3.13
5784	Repair of other fistula of bladder	8.78	6.19	73	440	3.13
8382	Graft of muscle or fascia	8.78	8.78	440	440	3.13
3794	Implantation or replacement of automatic cardioverter/defibrillator, total system [aicd]	8.75	5.25	38	432	3.12
3958	Repair of blood vessel with unspecified type of patch graft	8.72	8.72	421	421	3.10
8605	Incision with removal of foreign body or device from skin and subcutaneous tissue	8.70	8.70	415	415	3.09
3956	Repair of blood vessel with tissue patch graft	8.65	8.65	402	402	3.06
046	Transposition of cranial and peripheral nerves	8.56	8.56	378	378	3.02
8663	Full-thickness skin graft to other sites	8.56	5.81	56	378	3.02
3991	Freeing of vessel	8.49	8.49	360	360	2.98
3957	Repair of blood vessel with synthetic patch graft	8.48	8.48	358	358	2.98
3951	Clipping of aneurysm	8.48	8.48	357	357	2.97
0474	Other anastomosis of cranial or peripheral nerve	8.45	8.45	350	350	2.96
843	Revision of amputation stump	8.45	8.45	349	349	2.96
3959	Other repair of vessel	8.43	8.43	346	346	2.95
8080	Other local excision or destruction of lesion of joint, unspecified site	8.43	8.43	345	345	2.95
8683	Size reduction plastic operation	8.41	8.41	340	340	2.94
8609	Other incision of skin and subcutaneous tissue	8.39	8.39	335	335	2.93
0404	Other incision of cranial and peripheral nerves	8.35	8.35	327	327	2.91
3897	Central venous catheter placement with guidance	8.32	5.58	48	320	2.89
3925	Aorta-iliac-femoral bypass	8.32	8.32	320	320	2.89
8196	Other repair of joint	8.29	8.29	313	313	2.88
8381	Tendon graft	8.29	8.29	312	312	2.87
8383	Tendon pulley reconstruction other than hand	8.29	8.29	312	312	2.87

As before, *m* is the number of candidate codes in a map whereas *v* is the number of valid representations in a map. The *UR* measure (obtained by $\log _2(m)$) can directly be compared to *H*(*B*) (obtained by $\log _2(v)$). When $UR < H(B)$, *UR* has underestimated the expected coding complexity. When $UR > H(B)$, *UR* has overestimated the expected coding complexity. Otherwise, the measures are equal. The map id and description correspond to ICD-9-CM code and description, respectively

## Appendix E

### Implementation of Algorithm 4.1 using Python 3.6

To download the gem_i9pcs.txt file used in this code - this file represents the forward mappings from ICD-9-CM Vol.3 to ICD-10-PCS, go to https://www.cms.gov/Medicare/Coding/ICD10/2015-ICD-10-PCS-and-GEMs> *2015 General Equivalence Mappings (GEMs)—Procedure Codes and Guide (ZIP)* > *gem_i9pcs.txt*
